# The Physician’s Duty

**Published:** 1885-01

**Authors:** 


### THE PHYSICIAN'S DUTY.

In a press notice of the recent death of a distinguished member of the medical profession, the following is told:

A friend once remarked to him, "Doctor, what necessity is there for this ceaseless labor and study at your time of life?" With a look of astonishment, never to be forgotten, he replied: "My dear sir, I am under bonds to do it. When I offered my professional services to this community, there was an implied covenant on my part that, so far as God gave me strength and ability, I would use them for gathering up and digesting all that has been said or written in regard to the diseases to which human flesh is heir, and if I should lose a patient because of my ignorance of the latest and best experience of others in the treatment of a given case, a just God would hold me responsible for the loss, through inexcusable ignorance, of a precious human life; * * * and whenever I get my consent to be content with present professional attainments and trust my own personal experience for success, I will withdraw from practice and step from under a weight of honorable obligations which, with my best endeavors to meet them honestly and conscientiously, still sometimes is almost heavier than I can bear."

